# A Multi-Faceted Binding Assessment of Aptamers Targeting the SARS-CoV-2 Spike Protein

**DOI:** 10.3390/ijms25094642

**Published:** 2024-04-24

**Authors:** Laia Civit, Nima Moradzadeh, Anna Jonczyk, Patrick Neckermann, Benedikt Asbach, David Peterhoff, Ralf Wagner, Michael Famulok, Günter Mayer, Jørgen Kjems, Julián Valero

**Affiliations:** 1 Interdisciplinary Nanoscience Center, Aarhus University, DK-8000 Aarhus, Denmark; lcivit@inano.au.dk (L.C.); jk@mbg.au.dk (J.K.); 2 Life and Medical Sciences (LIMES), University of Bonn, 53121 Bonn, Germany; s0nimora@uni-bonn.de (N.M.);; 3 Institute of Medical Microbiology and Hygiene, Molecular Microbiology (Virology), Regensburg University, 93053 Regensburg, Germany; 4 Institute of Clinical Microbiology and Hygiene, University Hospital Regensburg, 93053 Regensburg, Germany; 5 Center of Aptamer Research & Development, University of Bonn, 53121 Bonn, Germany; 6 Department of Molecular Biology and Genetics, Aarhus University, DK-8000 Aarhus, Denmark

**Keywords:** aptamers, SARS-CoV-2, spike protein, COVID-19, comparative analysis

## Abstract

The COVID-19 pandemic has underscored the critical need for the advancement of diagnostic and therapeutic platforms. These platforms rely on the rapid development of molecular binders that should facilitate surveillance and swift intervention against viral infections. In this study, we have evaluated by three independent research groups the binding characteristics of various published RNA and DNA aptamers targeting the spike protein of the SARS-CoV-2 virus. For this comparative analysis, we have employed different techniques such as biolayer interferometry (BLI), enzyme-linked oligonucleotide assay (ELONA), and flow cytometry. Our data show discrepancies in the reported specificity and affinity among several of the published aptamers and underline the importance of standardized methods, the impact of biophysical techniques, and the controls used for aptamer characterization. We expect our results to contribute to the selection and application of suitable aptamers for the detection of SARS-CoV-2.

## 1. Introduction

COVID-19 has become one of the most devastating pandemics in the history of humankind and emphasizes the importance of developing diagnostic and therapeutic platforms that enable surveillance and rapid intervention against viral infections. Efforts to protect cells from viral infection have primarily focused on targeting the molecular mechanisms of virus–host cell interaction and infection. In the case of COVID-19, the SARS-CoV-2 virus utilizes the receptor binding domain (RBD) of the spike glycoprotein to bind to the ACE-2 receptor on host cells. This interaction is the initial step for viral entry and infection. mRNA vaccines have been developed as a prophylactic approach where the endogenous expression of spike protein-based antigens stimulates the production of antibodies [1,2]. Therapeutic approaches have focused on the development of molecular binding agents that specifically target the spike protein and can be used to antagonize the interaction with the ACE-2 receptor. Various protein-based binders, such as antibodies [3,4,5], small peptides [6,7,8], and de novo-designed proteins [9,10,11,12], have been reported to efficiently bind to the spike protein and block viral infection both in vitro and in vivo. These binders have also been implemented in standard detection assays, such as ELISA or lateral flow assays (LFA).

Aptamers, which are short single-stranded oligonucleotides displaying high affinity and specificity towards cognate targets and generated through a process termed SELEX (systematic evolution of ligands by exponential enrichment), have also been devised to bind the SARS-CoV-2 spike protein. Since the start of the COVID-19 pandemic, various RNA and DNA aptamers have been reported to target different epitopes of the SARS-CoV-2 spike protein, with most of them focusing on the receptor binding domain and S1 subunit [13,14,15,16,17]. We have contributed to the development of two aptamers targeting the spike protein, as reported in two independent publications. In Schmitz et al. [18], we used an automated selection procedure to identify a DNA aptamer, called SP6, that binds to the SARS-CoV-2 spike protein with low nanomolar affinity but not to RBD. This aptamer inhibits pseudovirus infection in cells, thus potentially providing a mechanism for virus neutralization independent of RBD. In Valero et al. [19], we described the selection of a 2’F-modified RNA aptamer, termed RBD-PB6, that binds to the RBD of the wild-type (WT) spike protein with a K_d_ of 18 nM. Engineering and multimerization of the aptamer enhanced its binding strength to the low picomolar range. Moreover, it showed efficient viral neutralization in vitro with an IC_50_ of 46 nM in a plaque reduction assay. In addition to these, several other aptamers were described to bind the spike glycoprotein from SARS-CoV-2, and some of them also revealed inhibitory potential. The pandemic therefore sparked the rapid identification of several aptamers for the same protein by independent groups in a yet unparalleled manner. This circumstance demonstrates the strength of the aptamer field’s ability to rapidly react to emerging threats, evidencing aptamer identification as a potential emergency discovery technology. Also, the availability of many different aptamers binding to the spike glycoprotein provides a unique possibility for conducting a comparative study. In this regard, we set out to test the binding characteristics of the different aptamers using complementary analysis platforms. A recent cross-comparative study assessed the affinity of different aptamers binding to the SARS-CoV-2 spike protein through filter retention analysis and showed consistent binding of all tested aptamers [20]. In contrast, our comparative study reveals clear differences and suggests that the specificity for some of the aptamers might be influenced by the experimental conditions and that their binding is primarily directed to associated purification tags rather than to the spike protein. We believe that the results of our study will contribute to the general assessment of aptamers, not only as SARS-CoV-2 detection reagents, and will also shed light on the importance of the choice of binding assay, experimental conditions, and the use of appropriate controls.

## 2. Results

For our comparative study, we selected ten aptamers from previous reports [13,14,15,16,17,18,19], most of them raised against the RBD domain of spike (CoV2-RBD-1C, CoV2-RBD-4C, CoV2-6C3, RBD-PB6, Aptamer-1 and 2), four towards the S1 subunit (nCoV-S1-A1 and 2, MSA1-T3 and MSA5-T4), and one against the full trimeric spike protein (SP6.41). Their specific sequences and targets used for selection can be found in Supplementary Table S1, and the results are summarized in Table 1. To ensure consistency, the aptamers tested in this study were assessed by three independent research laboratories: Kjems and Valero labs (biolayer interferometry (BLI) studies), Famulok and Mayer labs (enzyme-linked oligonucleotide assay (ELONA) and flow cytometry binding studies). All experiments were performed at single concentrations, approx. 10 times above the reported binding constants, to ensure signal detection.

**Figure 1:**
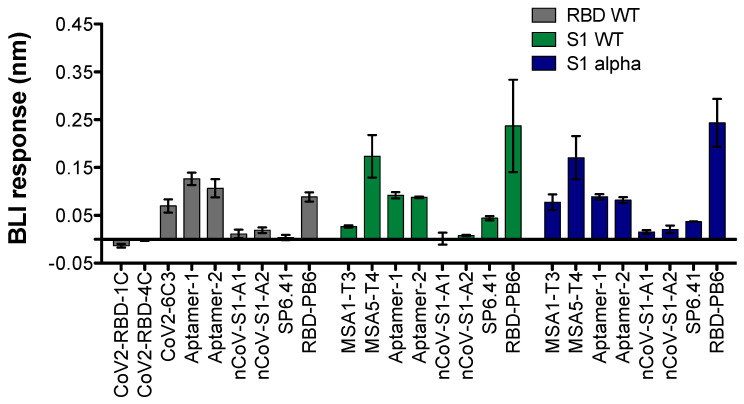
BLI screening of aptamers binding to their SARS-CoV-2 spike protein constructs (1 µM aptamer concentration and reported buffer conditions). Proteins: His-tagged RBD WT (grey bars), S1 WT (green bars), or S1 alpha (blue bars) constructs immobilized on Ni-NTA sensors (n = 2 singlets; mean ± SD). The BLI response is the result of a specific BLI signal after subtracting the buffer background signal at the end of the association phase (t = 195 s).

We first screened the binding parameters using biolayer interferometry. Refolding of the aptamer, buffer, and assay conditions were adapted as described in the respective original references (Table S2). First, the different aptamers were tested for binding to the original spike protein constructs used for selection (Figure 1). To that end, His-tagged spike RBD (WT) as well as the S1 subunit protein constructs (WT and alpha variants) were immobilized on a Ni-NTA BLI sensor. When immobilizing RBD, we observed binding with CoV2-6C3, Aptamer-1, Aptamer-2, and RBD-PB6, but we could not detect significant signals using CoV2-RBD-1C, CoV2-RBD-4C, and nCoV-S1-A1 and 2 (Figure 1, grey bars). We also tested the binding of aptamers to the S1 domain of the spike variants from the WT or alpha SARS-CoV-2 strains (Figure 1, green and blue bars, respectively). Upon immobilization of S1 proteins (WT and alpha variants), Aptamer-1 and 2, truncated versions of MSA1 and MSA5, as well as RBD-PB6, displayed significant binding signal intensity. As previously described, SP6.41 did not exhibit binding for RBD and only showed a close-to-background signal when tested against S1 variants, as it is reported to target the full spike protein.

**Figure 2:**
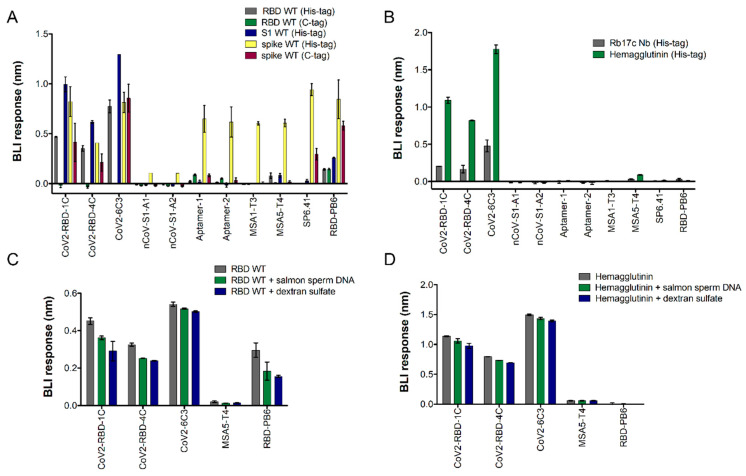
Comparative binding analysis of spike SARS-CoV-2 aptamers using biolayer interferometry (BLI): (**A**) BLI signal obtained for each aptamer targeting different SARS-CoV-2 spike proteins, including RBD WT His-tag (300 nM; grey bars) and RBD WT C-tag (300 nM; green bars); S1 WT His-tag (150 nM; blue bars); spike WT His-tag (100 nM; yellow bars); spike WT C-tag (100 nM; red bars); or (**B**) unrelated His-tagged proteins (hemagglutinin at 100 nM and Rb17c Nb at 500 nM). Biotinylated aptamers were immobilized on streptavidin-coated sensors. Binding was continuously monitored, and after 200 s of incubation with the corresponding proteins, aptamers were subsequently incubated in buffer without protein for the dissociation phase. Sensogram maximum intensities were plotted in bars (n = 2 singlets; mean ± SD). (**C**) Binding analysis with target RBD-his protein and hemagglutinin; and (**D**) in the presence or absence of salmon sperm DNA (0.1 mg/mL) and dextran sulfate (0.1 mg/mL) (n = 2 singlets; mean ± SD).

In an alternative assay configuration, biotinylated aptamers were immobilized on the surface of a streptavidin BLI sensor and tested against different spike protein constructs (Figure 2). For the binding assay, we employed the WT variant of the full SARS-CoV-2 trimeric spike protein, S1, and RBD subunits. To evaluate the effect of the protein purification tag and expression system on aptamer binding, we also used C-tag RBD and trimeric spike proteins, both expressed in insect S2 cells, instead of the mammalian HEK293 cell. We observed binding of the aptamers CoV2-RBD-1C, CoV2-RBD-4C, CoV2-6C3, and RBD-PB6, whereas Aptamers 1 and 2 only bound the His-tagged trimeric spike protein. Very low binding signals were observed using nCoV-S1-A aptamers with the His-tagged trimeric spike protein (Figure 2A). Truncated versions of the MSA aptamers (namely MSA1-T3 and MSA5-T4), reported to bind to different SARS-CoV-2 constructs, were also tested. Both aptamers bound the His-tagged spike WT protein. However, MSA1-T3 showed negligible binding to the other spike constructs, whereas MSA5-T4 displayed a low binding signal towards the His-tagged RBD and S1 but no binding to the C-tagged proteins. Aptamers CoV2-RBD-1C, CoV2-RBD-4C, and CoV2-6C3 showed positive binding to the trimeric C-tag protein but not to the RBD C-tag. Moreover, these aptamers showed strong signals to the smaller His-tagged proteins RBD and S1, in some cases, with a comparable response to the one observed for the trimeric spike proteins. In biolayer interferometry, the signal is proportional to the wavelength shift of white light, which depends not only on the binding strength but also on the size of the analyte. For RBD-PB6, we observed binding with all spike protein constructs independent of the tag used, and the BLI signal increased with the size of the analyte. Similarly, the SP6 aptamer variant SP6.41 displayed positive binding to His- and C-tagged spike proteins but not to RBD and S1 subunits, as these aptamers were reported to bind to the S2 spike subunit (Figure 2A). To study the specificity of these aptamers and exclude unspecific binding to the positively charged His-tag through electrostatic interactions, we used two His-tagged proteins unrelated to the SARS-CoV-2 spike, a nanobody (Rb17c, 15 kDa) [21] and hemagglutinin (A/England/195/2009 variant, 192 kDa), as controls. As shown in Figure 2B, the aptamers CoV2-RBD-1C, CoV2-RBD-4C, and CoV2-6C3 displayed a high BLI response in the presence of both His-tagged hemagglutinin and a nanobody. The signal was proportional to the size of the proteins, suggesting binding to the His-tag in the BLI setup. MSA5-T4 showed a lower binding response to the control proteins (Figure 2B) compared to CoV2-RBD-1C, CoV2-RBD-4C, and CoV2-6C3. All other aptamers did not show significant binding to His-tagged control proteins.

To corroborate that the interaction with the His-tag observed for aptamers CoV2-RBD-1C, CoV2-RBD-4C, and CoV2-6C3 is not an artifact due to unspecific electrostatic interactions, the binding assays were repeated in the presence or absence of salmon sperm DNA or dextran sulfate using His-tagged RBD protein and His-tagged hemagglutinin as controls. Salmon sperm DNA and dextran sulfate are widely used as unspecific competitors to study binding specificity as they bind non-specifically to positively charged protein subunits [22,23,24]. We observed that the BLI binding signal remains largely unaffected with or without competitors when using RBD (Figure 2C). A similar effect is observed when using hemagglutinin (Figure 2D). The PB6 aptamer showed consistent binding to RBD, with a slight reduction in binding response in the presence of competitors, probably due to competing electrostatic interactions, and no binding to hemagglutinin. 

Overall, using this specific setup, the BLI data indicate that some of the reported aptamers show binding only to His-tagged proteins, whereas other aptamers unexpectedly display weaker or no BLI signal to any of the spike protein constructs tested. However, many of the tested aptamers, except nCoV-S1-A aptamers, still show strong affinity to the His-tagged trimeric spike protein, comparable to the signal obtained with SP6 and RBD-PB6 aptamers.

**Figure 3:**
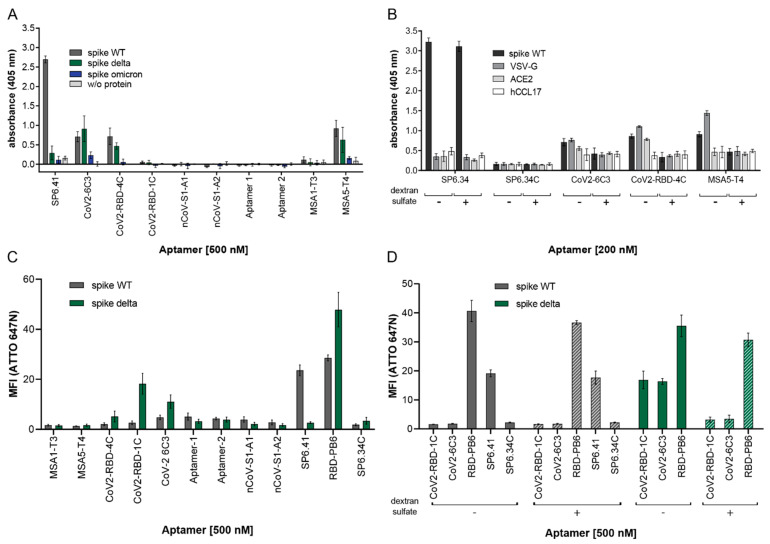
Comparative binding analysis of spike SARS-CoV-2 aptamers using enzyme-linked oligonucleotide assay (ELONA) (**A**,**B**) and flow cytometry (**C**,**D**): (**A**) Binding analysis of aptamers targeting SARS-CoV-2 spike protein against His-tagged WT, delta, and omicron trimeric constructs. The proteins were immobilized on hydrophobic plates and incubated with a 500 nM aptamer for 30 min at 25 °C in the reported selection buffer. (**B**) Aptamers that exhibited binding to spike protein in (**A**) were tested for binding to spike WT, VSV-G, ACE2, and the chemokine human CCL17. The protein was immobilized on hydrophobic plates and incubated with 200 nM aptamer for 30 min at 25 °C in the reported selection buffer. The samples were incubated either with or without 0.1 mg/mL dextran sulfate as a competitor. (**C**) Binding analysis of aptamers targeting His-tagged spike WT and delta trimeric constructs. Biotinylated aptamers were conjugated to the ATTO647N–Streptavidin bioreagent. The proteins were immobilized on Dynabeads™ His-tag isolation beads and incubated with 500 nM labeled oligonucleotides for 30 min at 25 °C (or 37 °C for SP6.41) in the respective binding buffers. (**D**) Interaction analysis of aptamers in the absence (plain-color bars) or presence (dashed bars) of dextran sulfate. (n = 2 duplicates, mean ± SD).

In addition to the BLI measurements, we also assessed the binding of the aptamers to the spike WT, delta, and omicron variants using an enzyme-linked oligonucleotide assay (ELONA). In this assay format, the proteins were coated on hydrophobic plates. The buffer and refolding conditions were used as described in the original publications of the respective aptamers (Table S2), and the aptamers were biotinylated to enable their detection with streptavidin–horseradish peroxidase (SA-HRP), and ABTS as substrate. The ELONA assays show that aptamers MSA5-T4, CoV2-RBD-4C, and CoV2-6C3 bind to the His-tagged spike WT and delta, while CoV2-6C3, MSA5-T4, and Sp6.41 showed close-to-background binding to the His-tagged omicron variant (Figure 3A). The two SP6 variants exclusively showed binding to spike WT, providing a strong signal of about 3-4-fold with respect to the other aptamers. To assess the influence of the His-tag on binding, the aptamers were also tested for binding to His-tag-bearing, non-spike proteins VSV-glycoprotein, ACE2, and the non-tagged small chemokine hCCL17 (Figure 3B). MSA5-T4 showed more intense binding towards VSV-G compared to spike WT protein but did not bind to His-tagged ACE2 and hCCL17. CoV2-RBD-4C showed similar binding to all three His-tagged proteins, while CoV2-6C3 displayed close-to-background-level binding to all the proteins independent of the presence of dextran sulfate. Except for SP6, the interaction of all three other aptamers (MSA5-T4, CoV2-6C3, CoV2-RBD-4C) with spike WT and the unrelated proteins was completely abolished by the addition of 0.1 mg/mL dextran sulfate as a competitor (Figure 3B). 

Finally, we also used flow cytometry-based binding assays to verify the BLI and ELONA results. For this, biotinylated aptamers were conjugated to ATTO647N–streptavidin to detect the interaction with trimeric His-tagged spike WT and delta proteins immobilized on NTA-magnetic beads (Figure 3C). The buffers described in the original publications for each aptamer were applied to the assay. All aptamers were refolded using the temperature ramp shown in Table S3. Initially, we performed the binding assay in the absence of Tween-20, as reported in the original publications (Figure S1). However, without Tween-20, we observed significant fluctuations and technical difficulties in terms of performance and reproducibility by flow cytometry (Figure S2). Tween-20 is usually added to reduce non-specific adsorption of the analyzed components to bead surfaces and to prevent aggregation of beads with the immobilized protein. In the presence of Tween-20, the mean fluorescence intensity of the beads carrying the spike WT demonstrated background intensity (MFI values < 5) similar to SP6.34C (non-binding control) for all aptamers tested, except for SP6.41 and RBD-PB6. (Figure 3C). Using the trimeric His-tagged spike delta protein revealed binding of CoV2-RBD-1C, CoV2-6C3, and RBD-PB6 and no binding to SP6.41, consistent with our previous findings (Figure 3C). 

The abundance of positively charged basic amino acids is significantly higher in the delta and omicron spikes compared to the WT [25,26]. This expands the electropositive surface of the delta variant and potentially enables higher unspecific interactions with the negatively charged aptamers. To investigate this charge effect on the binding of CoV2-RBD-1C and CoV2-6C3 aptamers to the delta variant further, we performed a flow cytometry binding assay in the presence of dextran sulfate, an anionic non-specific competitor (Figure 3D). We also included SP6.41 and RBD-PB6 to analyze the effect of the competitor on their binding properties. Dextran sulfate significantly reduced the interaction of CoV2-RBD-1C and CoV2-6C3 aptamers with the spike delta protein, whereas the binding of SP6.41 to the WT and of RBD-PB6 to both spike WT and delta constructs was not affected (Figure 3D). 

## 3. Discussion

In this study, we have analyzed a variety of aptamers reported to bind to the SARS-CoV-2 spike protein by using different techniques such as BLI, ELONA, and flow cytometry (summarized in Table 2). Several of these aptamers show variable behavior depending on different factors, including the different biophysical techniques, the design of the assay, and the use of competitors (e.g., salmon sperm DNA, dextran sulfate) or detergents (Tween-20). 

We observed unspecific binding of various aptamers to His-tagged proteins that are unrelated to SARS-CoV-2. Using BLI, where the protein was immobilized on the surface via its His-tag, we found no binding evidence for the aptamers CoV2-RBD-1C and CoV2-RBD-4C to the RBD domain. However, the immobilization of these aptamers and subsequent incubation with different proteins led to interaction signals for all His-tagged proteins tested, including a published nanobody (Rb17c) and hemagglutinin. Similar results were found when using the aptamer CoV2-6C3. This interaction profile was maintained in the presence of the non-specific competitor’s salmon sperm DNA and dextran sulfate. We observed a reduction in the interaction of CoV2-RBD-1C, CoV2-RBD-4C, and SP6.41 aptamers with the spike WT protein when changing the His-tag to a C-tag. These results can be explained either by differences in the glycosylation pattern and the folding of C-tag proteins due to their expression in insect cells or, more likely, by specific recognition of the His-tag. In contrast, the interaction with the spike protein was retained for CoV2-6C3 and RBD-PB6 and found to be independent of the nature of the tag. In the case of CoV2-6C3, a hybrid selection strategy using RBD domains expressed in HEK293 cells and insect cells was used, which might explain its binding behavior. Despite this, CoV2-6C3 does not show binding to the RBD WT C-tag. When immobilized on the BLI surface, aptamers MSA1-T3, MSA5-T4, and Aptamer-1 and -2 showed binding to the His-tagged spike WT protein but a low or negligible signal for the rest of the protein constructs. No interaction was observed for the aptamers nCoV-S1-A1 and -A2.

Using ELONA to assess the interaction properties of the aptamers, we observed moderate binding to the spike protein in the presence of dextran sulfate but also to other proteins, e.g., ACE2 or VSV-G, for the aptamers CoV2-RBD-4C, CoV2-6C3, and MSA5-T4. A spike-specific binding signal was detected for SP6 variants independent of the addition of dextran sulfate. Other aptamers tested showed negligible binding.

The addition of Tween-20 also had a major impact on the replicability of the results of the flow cytometric binding studies. Tween-20 is widely used to reduce the self-aggregation of functionalized beads in flow cytometry, improving assay readout and reproducibility. In its presence, the aptamers MSA5-T4, CoV2-RBD-1C, CoV2-RBD-4C, and CoV2-6C3 interestingly showed moderate to good binding exclusively to the spike delta, even though these aptamers were originally selected using the WT protein. Aptamer-2 displayed low-to-moderate binding to both protein variants, whereas the SP6.41 aptamer consistently showed affinity to the spike WT, independent of dextran sulfate, but no interaction with the spike delta. RBD-PB6 displayed strong interactions with both WT and delta spike proteins, independent of dextran sulfate.

Many of these aptamers were successfully used by other research groups on different biosensing platforms for the detection of the SARS-CoV-2 spike protein [27,28,29]. Recently, a comprehensive comparative analysis of different aptamers was published, many of which were also used in the present study [20]. The binding of these to different spike protein variants was determined and quantified only using dot-blot analyses. Several controls were used, including a control His-tagged protein. They found that all aptamers tested exhibited excellent binding affinity to the different SARS-CoV-2 variants used in the study. It was also found that the SP6 aptamer binds to the spike protein of omicron and delta variants with low nanomolar affinities. These findings could not be confirmed in our studies using three different binding methods. Moreover, we corroborated that SP6 does not bind to other variants of the spike protein (Figure 3D and Figure S2) and that the binding of RBD-PB6 to the spike omicron BA.2 variant is drastically reduced to background levels, probably due to the high number of mutations that this variant has in the RBD region [30].

## 4. Materials and Methods

All biotinylated and non-biotinylated DNA oligonucleotides were synthesized by Integrated DNA Technologies. RBD-PB6 aptamer was produced according to the original paper [19]. For BLI binding experiments, 96-well plates (black, flat bottom, Greiner, Kremsmünster, Austria) were used. Proteins used in this study are summarized in Table S4.

### 4.1. BLI Binding Assay

BLI binding experiments were performed on Octet RED96 equipment (ForteBio, Menlo Park, CA, USA) and analyzed using either the instrument’s software or Prism software (GraphPad Prism 5.0, GraphPad Software, Boston, MA, USA). All the data shown corresponds to the BLI signal at the end of the association phase (t = 195 s). In general, an orbital shake speed of 1000 rpm was used for BLI experiments.

For initial screening of the SARS-CoV-2-published aptamers, different His-tagged spike protein constructs (RBD WT [31] and S1 WT or alpha (SinoBiological, Beijing, China)) were diluted in binding buffer (50 mM HEPES pH 7.4, 6 mM KCl, 150 mM NaCl, 2.5 mM CaCl_2_, 2.5 mM MgCl_2_ for MSA aptamers, or PBS pH 7.4, 1 mM MgCl_2_ for the rest of the aptamers, both supplemented with 0.1 mg/mL BSA and 0.02% Tween-20) at 2.5 µg/mL concentration and immobilized onto Ni-NTA-coated biosensors (OCTET Ni-NTA [NTA] Biosensors, Sartorius, Goettingen, Germany). Aptamers were screened at 1 μM concentration (previously folded in corresponding buffer according to Table S2). Baseline was recorded to establish initial BLI signals prior to each binding event (including association, dissociation, and regeneration steps). First, the protein-coated sensor was dipped into the aptamer solution for 200 s (association step) and then into buffer only for 100 s (dissociation step). Finally, three cycles of regeneration were performed, consisting of first dipping the sensor into a glycine solution (10 mM at pH 1.4) and then into buffer for 5 s each. The process was repeated for each aptamer tested.

Pre-folded biotinylated aptamers diluted in proper binding buffer at 80 nM concentration were loaded on streptavidin-coated sensors (OCTET Streptavidin [SA] Biosensors, Sartorius, Goettingen, Germany) until a response of 0.2 nm was reached. The baseline was recorded prior to binding measurements. Sensors were then dipped in the different tested proteins (SARS-CoV-2-related proteins and control proteins) at fixed concentrations for 200 s during the association step. Then, sensors were dipped in buffer only for 100 s for the dissociation step. Regeneration was performed by three cycles of consecutive steps, first dipping into phosphoric acid (500 mM) and then into binding buffer for 5 s each. Subsequently, the aptamer-loaded sensor was dipped again in the next protein for another binding measurement.

### 4.2. Enzyme-Linked Oligonucleotide Assay 

For immobilization on hydrophobic plates, the proteins were diluted to 3 µg/mL in PBS, and 20 µL of dilution per well was immobilized overnight at 4 °C on a 96-well half-area plate (Greiner Bio). Free protein was removed by washing three times with 100 µL of PBS with 0.05% Tween-20, and the wells were blocked with 5% BSA in PBS for 30 min at 4 °C and an additional 30 min at room temperature. Wells were washed once with the respective binding buffer of each aptamer (Table S2), and 20 µL of biotinylated aptamer diluted to the stated concentration was added to each well and incubated for 30 min at 25 °C. Unbound DNA was removed by washing three times with 100 µL of the binding buffer of the respective aptamer. A 1:1000 dilution of streptavidin–horseradish peroxidase (SA-HRP) solution (GE Healthcare, Alger, OH, USA) in the respective binding buffer was prepared and 20 µL added into each well. After 30 min of incubation at room temperature, the wells were washed twice with 100 µL of washing buffer. The final protein-DNA-SA-HRP complex was incubated with 50 µL of ABTS substrate (Thermo Fisher, Waltham, MA, USA) for 30 min at room temperature. The absorbance at 405 nm was measured with a Tecan NanoQuant plate reader (Tecan Group Ltd., Männedorf, Switzerland). 

### 4.3. Flow Cytometry-Based Assay

To enable interaction analysis via flow cytometry, biotinylated aptamers were incubated at RT for 30 min with ATTO647N–Streptavidin (ATTO647N-SA) conjugate at a 1:1 molar ratio. The concentration of oligonucleotides after refolding was measured using absorbance at 260 nm, and the final concentration of the labeled biotinylated aptamers was calculated, considering the dilution factor, after the addition of the dye conjugate. In-house expressed and purified 8× His-tagged trimeric SARS-CoV-2 spike protein variants (wild-type and delta) were thawed on ice, then centrifuged at 20,000× *g* for 5 min at 4 °C to pellet any aggregates. The protein concentration in the supernatant was measured using the molar extinction coefficient of the respective protein construct using absorbance at 280 nm on a Nanodrop 2000C (Thermo Fisher Scientific). Dynabeads™ His-Tag Isolation and Pulldown (Thermo Fisher Scientific) was the matrix of choice to immobilize the proteins via 8× His. Initially, the appropriate volume of beads (1 µL of bead stock for 48 pmol of spike protein) was transferred to a 1.5 mL Eppendorf tube and diluted 1:10 in immobilization buffer (50 mM NaH_2_PO_4_, 300 Mm NaCl_2_, 0.02% Tween-20, pH 8.0). The beads were separated on DynaMag™-2 (Thermo Fisher Scientific) and washed three times with the immobilization buffer. Appropriate amounts of each protein were incubated at 4 °C for 1 h with the beads in immobilization buffer. Afterwards, the beads were washed with PBS (137 mM NaCl, 3.9 mM Na_2_HPO_4_, 2.7 mM KCl, and 1.5 mM KH_2_PO_4_ in ddH_2_O). The binding was performed by preparing a binding solution comprising 4.5 µL of 2× binding buffer (Table S3), 500 nM labeled aptamer, and 1 uL of the bead–protein solution in a total volume of 10 µL. The aptamers and proteins were incubated for 30 min at 25 °C (or 37 °C for SP6.41 and 34C) and then washed with 100 µL of binding buffer two times in the presence of Tween-20. The fluorescence intensity of ATTO647N in the beads was measured by flow cytometry. 

## 5. Conclusions

In conclusion, our study reveals inconsistencies in the claimed target specificity and affinity among several of the published aptamers. We also describe that despite RBD-PB6 and SP6.41 aptamers are consistently binding to different WT spike protein constructs, they do not bind to spike omicron variants in any of the reported conditions. This contradicts previous reports describing the binding of SP6 to other spike proteins, including delta and omicron variants [20]. It has previously been reported that specific aptamers may behave differently depending on the biophysical technique used for their characterization [32,33] or when different blocking agents are applied to avoid unspecific interactions [34]. The discrepancy may in part be explained by the techniques used (BLI, ELONA, and flow cytometry versus dot plotting and others), experimental conditions, controls used, and protein sources, but also potential binding to tags remaining in the purified protein appears to explain some of the results. We acknowledge the complexity of our comparative study and that aptamer binding characteristics depend, among others, on many factors such as pH, buffer, aptamer folding, and differences in the target or the biophysical technique used for binding characterization. Therefore, there might be discrepancies attributed to the lab variations for different experimental conditions applied. However, we expect our data to shed light on the applicability of the different aptamers tested in this study and to contribute to the selection of suitable aptamers for future applications, including the detection of SARS-CoV-2 viruses. The findings of previous studies and the results shown here strongly advocate for standardized approaches to evaluate aptamer target interactions, e.g., the use of competitors, appropriate non-binding control sequences, and target proteins with different or no tags, as described in a recently published white paper [35]. For reliable aptamer validation, it is also recommended to employ different binding techniques and setups that offer a substantial difference for target display or mode of detection and cross-validate the findings, as well as to properly characterize and describe the buffer and ion concentration dependence for aptamer folding and function.

## Figures and Tables

**Table 1:** Summary of the binding techniques and affinity (K_D_) reported of the SARS-CoV-2 spike aptamers used in this comparative study.

Name	Target	Analytical Technique	Binding Affinity (K_D_)	Reference
CoV2-RBD-1C	RBD	Flow cytometry	5.8 ± 0.8 nM	[15]
CoV2-RBD-4C	RBD		19.9 ± 2.6 nM	
CoV2-6C3	RBD	Flow cytometry	44.78 ± 9.97 nM	[16]
Aptamer-1	RBD	Flow cytometry	6.05 ± 2.05 nM	[14]
Aptamer-2	RBD		6.95 ± 1.1 nM	
nCoV-S1-A1	S1	Capillary electrophoresis	~0.327 ± 0.016 nM (S1) 1.56 ± 0.22 μM (RBD)	[17]
nCoV-S1-A2	S1		0.313 ± 0.078 nM	
MSA1-T3	S1	Dot-blot	3.1 ± 0.4 nM	[13]
MSA5-T4	S1		6.3 ± 0.8 nM	
SP6.41	S	Surface plasmon resonance	13.9 ± 0.6 nM for full-length SP6 (not specified for truncated SP6.41)	[18]
RBD-PB6	RBD	Biolayer Interferometry	~18 nM	[19]

**Table 2:** Summary of binding assays performed with SARS-CoV-2 aptamers.

Aptamer	BLI	ELONA	Flow Cytometry
CoV2-RBD-1C	No binding to His-immobilized RBD protein. Binds to His-tag spike and control proteins in solution, but not to C-tag spike proteins. Insensitive to competitors.	No binding to His-tag trimeric spike protein coated on plates.	No binding to the WT trimeric spike. Moderate binding to the delta variant; decreases in the presence of dextran sulfate.
CoV-2-RBD-4C	No binding to His-immobilized RBD protein. Binds to His-tag spike and control proteins in solution, but not to C-tag spike proteins. Insensitive to competitors.	Binds to His-tag WT and delta spike protein coated on plates. Binding to His-tag VSV-glycoprotein and ACE2. No binding in presence of dextran sulfate.	No binding to the WT spike. Large variation in binding to the WT spike in the absence of T-20. Weak binding to the delta variant.
CoV2-RBD-6C3	Binds to His-immobilized RBD protein. Binds to His-tag spike and control proteins in solution. Binding to C-tag spike, but not to C-tag RBD. Insensitive to competitors.	Binds to His-tag WT, delta, and omicron spike-coated on plates. Binding to His-tag VSV-glycoprotein. No binding in the presence of dextran sulfate.	Weak binding to the WT spike. Moderate binding to the delta variant; lost using dextran sulfate.
Aptamer-1	Binds to His-immobilized RBD and S1 proteins. Binds only to His-tag trimeric spike in solution. No binding with control His-tag proteins. No binding with C-tag spike proteins.	No binding to His-tag spike coated on plates.	Negligible binding to the WT and delta spike regardless of T-20. Negligible binding to the delta spike regardless of T-20.
Aptamer-2	Binds to His-immobilized RBD and S1 proteins. Binds only to His-tag trimeric spike in solution. No binding with control His-tag proteins. No binding with C-tag spike proteins.	No binding to His-tag spike coated on plates.	Negligible binding to the WT with T-20. Negligible binding to the delta regardless of T-20.
nCoV-S1-A1	Negligible binding for all protein constructs.	No binding to His-tag spike coated on plates	Negligible binding to the WT with T-20. Negligible binding to the delta regardless of T-20.
nCoV-S1-A2	Negligible binding for all protein constructs.	No binding to His-tag spike coated on plates.	Negligible binding to the WT with T-20. Negligible binding to the delta regardless of T-20.
MSA1-T3	Binds to His immobilized S1 alpha, but not to WT. Binds to His-tag WT spike protein in solution, but not to RBD or S1. No binding to C-tag and control proteins.	Negligible binding to His-tag spike coated on plates.	Large variation in binding characteristic of the WT spike in the absence of T-20. Negligible binding to the WT with T-20. Negligible binding to the delta regardless of T-20.
MSA5-T4	Binds to His immobilized S1 WT and alpha variants. Binds to His-tag WT spike protein in solution, but not to RBD or S1. No interaction with C-tag and control proteins. Insensitive to competitors.	Binds to His-tag WT and delta spike coated on plates. Interacts with His-tag VSV-G. No binding in presence of dextran sulfate.	Negligible binding to the WT and delta.
SP6.41	Binds to His-tag and C-tag WT spike protein in solution. No interaction with control proteins.	Binds to His-tag WT trimeric spike protein coated on hydrophobic plates. No binding to control proteins. Insensitive to competitor dextran sulfate.	Binds to the WT spike, insensitive to dextran sulfate. Negligible binding to the delta and omicron
RBD-PB6	Binds to His immobilized RBD and S1 (WT and alpha) variants. Binds to all His-tag and C-tag WT spike protein constructs (including RBD and S1) in solution. No binding to control proteins. Small decrease in signal with competitors.		Binds to the WT and delta spike, insensitive to dextran sulfate.

## Data Availability

The datasets obtained and analyzed in this study are available from the corresponding authors upon request.

## Supplementary Materials

The following supporting information can be downloaded at: https://www.mdpi.com/article/10.3390/ijms25094642/s1.
