# Supplementary material for: A Multi-Faceted Binding Assessment of Aptamers Targeting the SARS-CoV-2 Spike Protein
Source: Int J Mol Sci. 2024 Apr 24;25(9):4642. doi: 10.3390/ijms25094642 (PMC11083479; doi:10.3390/ijms25094642)
Supplement: Supplementary file 1 [file ijms-25-04642-s001.zip › ijms-2924353-supplementary.pdf]

Supporting information

A Multi-Faceted Binding Assessment of Aptamers Targeting the SARS-CoV-2 Spike Protein

Laia Civit [1,†], Nima Moradzadeh [2,†], Anna Jonczyk [2], Patrick Neckermann [3], Benedikt Asbach [3], David Peterhoff [3,4], Ralf Wagner [3,4], Michael Famulok [2,5], Günter Mayer [2,5,*,‡], Jørgen Kjems [1,6,‡] and Julián Valero [1,6,*,‡]

1 Interdisciplinary Nanoscience Center, Aarhus University, DK-8000 Aarhus, Denmark; lcivit@inano.au.dk (L.C.); jk@mbg.au.dk (J.K.)
2 Life and Medical Sciences (LIMES), University of Bonn, 53121 Bonn, Germany; s0nimora@uni-bonn.de (N.M.); ajonczyk@uni-bonn.de (A.J.); m.famulok@uni-bonn.de (M.F.)
3 Institute of Medical Microbiology and Hygiene, Molecular Microbiology (Virology), Regensburg University, 93053 Regensburg, Germany; patrick.neckermann@klinik.uni-regensburg.de (P.N.); benedikt.asbach@klinik.uni-regensburg.de (B.A.); david.peterhoff@klinik.uni-regensburg.de (D.P.); ralf.wagner@klinik.uni-regensburg.de (R.W.)
4 Institute of Clinical Microbiology and Hygiene, University Hospital Regensburg, 93053 Regensburg, Germany
5 Center of Aptamer Research & Development, University of Bonn, 53121 Bonn, Germany
6 Department of Molecular Biology and Genetics, Aarhus University, DK-8000 Aarhus, Denmark
* Correspondence: gmayer@uni-bonn.de (G.M.); jvalero@inano.au.dk (J.V.)
† These authors contributed equally to this work.
‡ These authors also contributed equally to this work.

Table S1. List of aptamers

Name	Sequence	Selection target	Reference
CoV2-RBD-1C	CAG CAC CGA CCT TGT GCT TTG GGA GTG CTG GTC CAA GGG CGT TAA TGG ACA	RBD	[2]
CoV2-RBD-4C	ATC CAG AGT GAC GCA GCA TTT CAT CGG GTC CAA AAG GGG CTG CTC GGG ATT GCG GAT ATG GAC ACG T	RBD	
CoV2-6	ATC CAG AGT GAC GCA GCA CCC AAG AAC AAG GAC TGC TTA GGA TTG CGA TAG GTT CGG GGG ACA CGG TGG CTT AGT A	RBD	[3]
CoV2-6C3	CGC AGC ACC CAA GAA CAA GGA CTG CTT AGG ATT GCG ATA GGT TCG G	RBD	
Aptamer-1	AT CCA GAG TGA CGC AGC ATC GAG TGG CTT GTT TGT AAT GTA GGG TTC CGG TCG TGG GTT GGA CAC GGT GGC TTA GT	RBD	[4]
Aptamer-2	AT CCA GAG TGA CGC AGC AAT TAC CGA TGG CTT GTT TGT AAT GTA GGG TTC CGT CGG ATT GGA CAC GGT GGC TTA GT	RBD	
nCoV-S1-A1	AG CAG CAC AGA GGT CAG ATG CCG CAG GCA GCT GCC ATT AGT CTC TAT CCG TGA CGG TAT GCC TAT GCG TGC TAC CGT GAA	S1	[5]
nCoV-S1-A2	AG CAG CAC AGA GGT CAG ATG GCA GCT AAG CAG GCG GCT CAC AAA ACC ATT CGC ATG CGG CCC TAT GCG TGC TAC CGT GAA	S1	

MSA1-T3	TC CGG TTA ATT TAT GCT CTA CCC GTC CAC CTA CCG GA	S1	[6]
MSA5-T4	CT TCC ACG GGT TTG GCG TCG GGC CTG GCG GGG GGA TAG TGC GGT GGA AG	S1	
SP6.41	CAA CCC ATG GTA GGT ATT GCT TGG TAG GGA TAG TGG GCT TG	S	[7]
SP6.34C	CCC ATG GTA GGT ATT GCT TGG TAG CGA TAG TGG G	Control sequence	
RBD-PB6	GGG GCC ACC AAC GAC AUU UGU AAU UCC UGG ACC GAU ACU UCC GUC AGG ACA GAG GUU GAU AUA AAU AGU GCC CAU GGA UCC	RBD	[1]

Table S2: Modifications, buffers and refolding conditions of the tested published aptamers via BLI and ELONA.

**Aptamer**	**Modification**	**Binding Buffer**	**Refolding**
MSA1-T3	w/wo 5'-Biotin	30 mM HEPES, 6 mM KCl, 150 mM NaCl, 2.5 mM $CaCl_2$, 2.5 mM $MgCl_2$	5 min 90°C and 20 min RT
MSA5-T4			
CoV2-RBD-1C	w/wo 5'-Biotin	PBS with 1 mM $MgCl_2$	5 min 90°C, 5 min ice and 10 min RT
CoV2-RBD-4C			
CoV2-6C3	w/wo 5'-Biotin	PBS with 1 mM $MgCl_2$	5 min 90°C, 5 min ice and 10 min RT
Aptamer-1	w/wo 5'-Biotin	PBS with 1 mM $MgCl_2$	Not specified; 5 min 90°C, 5 min ice and 10 min RT
Aptamer-2			
nCoV-S1-A1	w/wo 5'-Biotin	PBS with 1 mM $MgCl_2$	5 min 90°C and slow cooling to RT
nCoV-S1-A2			
SP6.41	w/wo 5'-Biotin	PBS with 3 mM $MgCl_2$	5 min 95°C
SP6.34c			
RBD-PB6	w/wo 3'-Biotin	PBS with 1 mM $MgCl_2$	2 min 90°C, 3 min 65°C,3 min 37°C, and cooling to RT

Table S3: Modifications, buffers and refolding conditions of the tested published aptamers via flow cytometry. SA: Streptavidin

Aptamer	Modification	Binding Buffer	Refolding
MSA1-T3	Biot/SA-ATTO647N	30 mM HEPES, 6mM KCl, 150 mM NaCl, 2.5 mM CaCl2, 2.5 mM $MgCl_2$	5 min 90°C and 10 min RT
MSA5-T4			
CoV2-RBD-4C	Biot/SA-ATTO647N	PBS with 1 mM $MgCl_2$	5 min 90°C and 10 min RT
CoV2-RBD-1C			
CoV2-6C3	Biot/SA-ATTO647N	PBS with 1 mM $MgCl_2$	5 min 90°C and 10 min RT
Aptamer-1	Biot/SA-ATTO647N	PBS with 1 mM $MgCl_2$	5 min 90°C and 10 min RT
Aptamer-2			
nCoV-S1-A1	Biot/SA-ATTO647N	PBS with 1 mM $MgCl_2$	5 min 90°C and 10 min RT
nCoV-S1-A2			
RBD-PB6	Biot/SA-ATTO647N	PBS with 1 mM $MgCl_2$	5 min 90°C and 10 min RT
SP6.41	Biot/SA-ATTO647N	PBS with 3 mM $MgCl_2$	5 min 90°C and 10 min RT
SP6.34C			

Table S4: SARS-CoV-2 related proteins and control proteins used.

Protein	Amino acids	Tag	Reference/supplier
SARS-CoV-2 RBD WT	319 to 532	His	[8]
SARS-CoV-2 RBD	319-591	Ctag	Cat. Num.: S2-45A-001 ExpreS2ion Biotechnologies
SARS-CoV-2 S1 WT	16-685	His	Cat. Num.: 40591-V08H SinoBiological
SARS-CoV-2 S1 B.1.1.7 (alpha)	1-685	His	Cat. Num.: 40591-V08H12 SinoBiological
SARS-CoV-2 Spike WT	1-1208	His	[8]
SARS-CoV-2 Spike WT	1-1208	His & TwinStrep	[7]
SARS-CoV-2 Spike WT	16-1208	Ctag	Cat. Num.: S2-46A-001 ExpreS2ion Biotechnologies
SARS-CoV-2 Spike Omicron BA.2		His	[8]
Hemagglutinin		His	Wagner lab
Rb17c Nanobody		His	[9]

Supporting figures

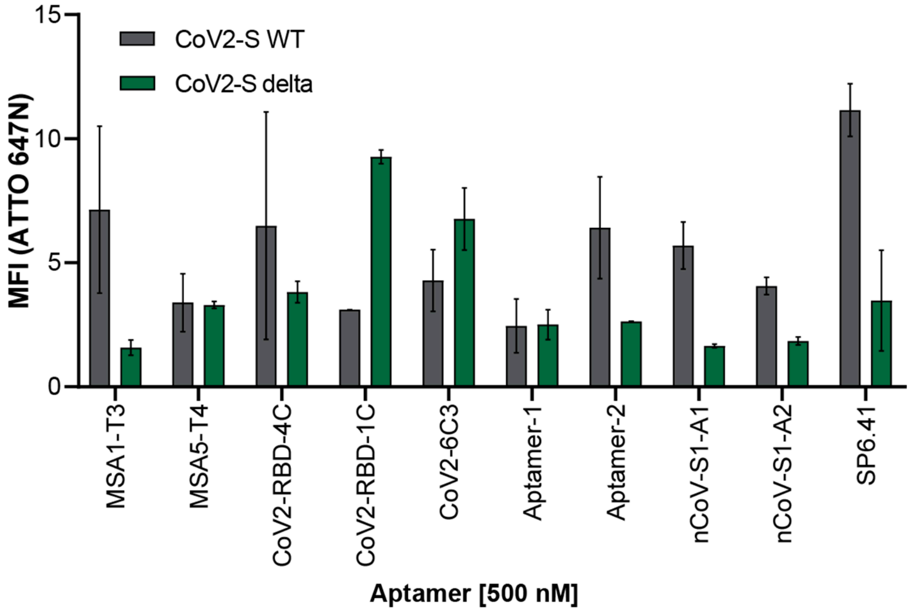

Figure S1: Binding analysis of aptamers targeting spike SARS-CoV-2 protein against wild-type (WT) and delta trimeric constructs via flow cytometry. Biotinylated aptamers were conjugated to ATTO647N-Streptavidin bioreagent. The proteins were immobilized on Dynabeads™ His-Tag Isolation and Pulldown (Invitrogen) and incubated with 500 nM labelled oligonucleotide for 30 mins at 25 °C (or 37 °C for SP6.41) in the respective binding buffers lacking Tween-20. (n=1 duplicates, mean ± SD).

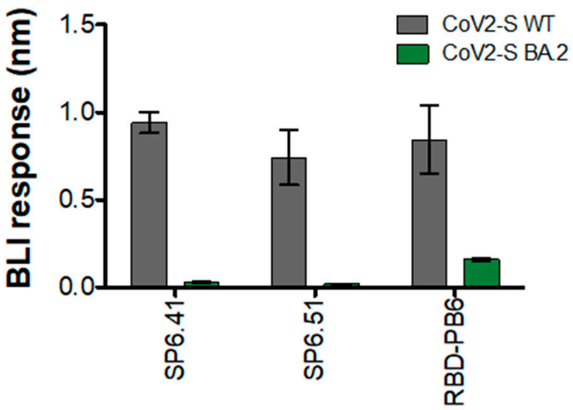

Figure S2: BLI binding analysis of SP6 variants and RBD-PB6 aptamers towards WT and omicron BA.2 spike protein variants. In these experiments immobilized aptamers were tested against the two protein variants at 100 nM each (n = 2, mean ± SD).

References

1. Valero, J.; Civit, L.; Dupont, D.M.; Selnihhin, D.; Reinert, L.S.; Idorn, M.; Israels, B.A.; Bednarz, A.M.; Bus, C.; Asbach, B.; et al. A serum-stable RNA aptamer specific for SARS-CoV-2 neutralizes viral entry. *Proceedings of the National Academy of Sciences* **2021**, *118*, e2112942118, doi:doi:10.1073/pnas.2112942118.
2. Song, Y.; Song, J.; Wei, X.; Huang, M.; Sun, M.; Zhu, L.; Lin, B.; Shen, H.; Zhu, Z.; Yang, C. Discovery of Aptamers Targeting the Receptor-Binding Domain of the SARS-CoV-2 Spike Glycoprotein. *Analytical Chemistry* **2020**, *92*, 9895-9900, doi:10.1021/acs.analchem.0c01394.
3. Sun, M.; Liu, S.; Wei, X.; Wan, S.; Huang, M.; Song, T.; Lu, Y.; Weng, X.; Lin, Z.; Chen, H.; et al. Aptamer Blocking Strategy Inhibits SARS-CoV-2 Virus Infection. *Angewandte Chemie International Edition* **2021**, *60*, 10266-10272, doi:https://doi.org/10.1002/anie.202100225.
4. Liu, X.; Wang, Y.-l.; Wu, J.; Qi, J.; Zeng, Z.; Wan, Q.; Chen, Z.; Manandhar, P.; Cavener, V.S.; Boyle, N.R.; et al. Neutralizing Aptamers Block S/RBD-ACE2 Interactions and Prevent Host Cell Infection. *Angewandte Chemie International Edition* **2021**, *60*, 10273-10278, doi:https://doi.org/10.1002/anie.202100345.
5. Yang, G.; Li, Z.; Mohammed, I.; Zhao, L.; Wei, W.; Xiao, H.; Guo, W.; Zhao, Y.; Qu, F.; Huang, Y. Identification of SARS-CoV-2-against aptamer with high neutralization activity by blocking the RBD domain of spike protein 1. *Signal Transduction and Targeted Therapy* **2021**, *6*, 227, doi:10.1038/s41392-021-00649-6.
6. Li, J.; Zhang, Z.; Gu, J.; Stacey, H.D.; Ang, J.C.; Capretta, A.; Filipe, C.D.M.; Mossman, K.L.; Balion, C.; Salena, B.J.; et al. Diverse high-affinity DNA aptamers for wild-type and B.1.1.7 SARS-CoV-2 spike proteins from a pre-structured DNA library. *Nucleic Acids Res* **2021**, *49*, 7267-7279, doi:10.1093/nar/gkab574.
7. Schmitz, A.; Weber, A.; Bayin, M.; Breuers, S.; Fieberg, V.; Famulok, M.; Mayer, G. A SARS-CoV-2 Spike Binding DNA Aptamer that Inhibits Pseudovirus Infection by an RBD-Independent Mechanism**. *Angewandte Chemie International Edition* **2021**, *60*, 10279-10285, doi:https://doi.org/10.1002/anie.202100316.
8. Peterhoff, D.; Glück, V.; Vogel, M.; Schuster, P.; Schütz, A.; Neubert, P.; Albert, V.; Frisch, S.; Kiessling, M.; Pervan, P.; et al. A highly specific and sensitive serological assay detects SARS-CoV-2 antibody levels in COVID-19 patients that correlate with neutralization. *Infection* **2021**, *49*, 75-82, doi:10.1007/s15010-020-01503-7.
9. Vaneycken, I.; Devoogdt, N.; Van Gassen, N.; Vincke, C.; Xavier, C.; Wernery, U.; Muyldermans, S.; Lahoutte, T.; Caveliers, V. Preclinical screening of anti-HER2 nanobodies for molecular imaging of breast cancer. *The FASEB Journal* **2011**, *25*, 2433-2446, doi:https://doi.org/10.1096/fj.10-180331.
